# Supplementary material for: Nicarbazin Residue in Tissues from Broilers Reared on Reused Litter Conditions
Source: Animals (Basel). 2022 Nov 10;12(22):3107. doi: 10.3390/ani12223107 (PMC9686525; doi:10.3390/ani12223107)
Supplement: Supplementary file 1 [file animals-12-03107-s001.zip › animals-1922408-supplementary.pdf]

# Nicarbazin Residue in Tissues from Broilers Reared on Reused Litter Conditions

**Vivian Feddern [1,*], Gerson N. Scheuermann [1], Arlei Coldebella [1], Vanessa Gressler [1], Gizelle C. Bedendo [2], Luizinho Caron [1], Antonio C. Pedroso [3], Danniele M. Bacila [4] and Anildo Cunha, Jr. [1]**

[1] Embrapa Suínos e Aves [Embrapa Swine and Poultry], BR 153, km 110, Concórdia 89715-899, SC, Brazil
[2] Embrapa Solos [Embrapa Soil], Setor de Transferência de Tecnologia, Jardim Botânico 1024, Rio de Janeiro 22460-000, RJ, Brazil
[3] Instituto Federal Catarinense, Concórdia 89703-720, SC, Brazil
[4] Departamento de Engenharia Química, Universidade Federal do Paraná, Curitiba 80060-000, PR, Brazil
* Correspondence: vivian.feddern@embrapa.br; Tel.: +55-49-3441-0400

## Supplementary material

### Materials and methods

### DNC Determination in Feed, Breast fillet, Liver and Poultry litter

#### Reagents

LiChrosolv® acetonitrile (ACN) was purchased from Merck (Darmstadt, Hessen, Germany). Methanol (MeOH, HPLC grade, ≥ 99.9%), *N,N*-dimethyl formamide (DMF, HPLC grade, ≥ 99.9%), and the analytical standards (Nicarbazin and DNC-$d_8$, both Vetranal®) were acquired from Sigma-Aldrich Co. (St Louis, MO, USA). Formic acid as eluent additive for LC-MS was purchased from Scharlab S. L. (Sentmenat, Spain). Ammonium acetate ($NH_4OAc$, 97%) and anhydrous sodium sulphate ($Na_2SO_4$, 99%) were obtained from Panreac (Barcelona, Spain) and Alphatec (S. J. dos Pinhais, PR, Brazil), respectively. Ultra-pure water (18 MΩ.cm at 25 °C) was produced in a Milli-Q® Advantage A10® system (EMD Millipore Corporation, Billerica, MA, USA).

#### Solutions

Nicarbazin and DNC-$d_8$ analytical solutions were prepared as described by Coleman et al. [3]. Nicarbazin (as DNC): stock at 1000 µg/mL (in DMF); intermediate at 10 µg/mL (in ACN); and standards at 25, 50, 125, 500, 1250, and 2500 ng/mL (in ACN). DNC-$d_8$ (used as internal standard): stock at 1000 µg/mL (in DMF); and internal standard solution (IS) at 1 µg/mL (in ACN).

**Feed**

The extraction procedure for feed was modified from elsewhere [1]. Ground sample (2.0 g) was weighed into a 50 mL conical polypropylene tube. After the addition of 90:10 (v/v) ACN:water (20 mL), the tube was subjected to rapid vortexing and then left for 30 min under rotary shaking. The solid was settled by centrifugation at 3,500*g* (5 min, 15 °C). A supernatant aliquot (2 mL) was applied to an SPE-C18 cartridge (500 mg, 3 mL) previously conditioned with 90:10 (v/v) ACN:water (2 x 2 mL). The supernatant percolated through the cartridge by gravity, and the eluate was received in a 15 mL conical polypropylene tube. Next, the cartridge was eluted slowly with 90:10 (v/v) ACN:water (2 x 2 mL), collecting the eluate cumulatively into the same 15 mL tube. The cartridge was drained under a vacuum. The eluate (about 7 mL) was evaporated until 2 mL under gentle $N_2$ flow at 45 °C. The sample extract was vortexed and transferred into a 2 mL vial for HPLC-UV analysis within 24 h. Aliquots of Nicarbazin stock solution (at 1000 mg/L) were added on blank feed samples for preparation of matrix-matched analytical curve at 6 DNC levels (10, 25, 50, 100, 150, and 200 mg/kg). These spiked samples were subjected to extraction as well. Blank feed fortified with DNC at 100 mg/kg was used as quality control sample (QCs) within every analyzed batch.

**Breast fillet and Liver**

DNC determination followed the procedure established under our laboratory conditions [2] which in turn is based on the method of Coleman et al. [3]. The freeze-dried sample (about 1.5 g of breast or 0.3 g of liver, equivalent to 5.0 and 1.0 g on a wet basis, respectively) was weighed into a 50 mL conical polypropylene tube and fortified with internal standard solution (200 µL). Subsequently, anhydrous $Na_2SO_4$ (1.0 g) was added and incorporated into the sample by vortex stirring. After the addition of ACN (20 mL), the tube was subjected to rapid vortexing and then left for 30 min under rotary shaking. The solid was settled by centrifugation at 3,500*g* (10 min, 5 °C). The supernatant was transferred to another 50 mL conical tube, and the pellet re-extracted with ACN (20 mL) following the same steps as before. The supernatants were combined for further volume adjustment to 50 mL with ACN. Based on Coleman et al. [3], nicarbazin standard solutions were added to blank sample extracts (either breast or liver) to prepare matrix-matched analytical curves containing the internal standard. Sample extract was filtered on a hydrophilic membrane (0.22 µm) into a 2 mL vial for LC-MS/MS determination within 48 h. Blank matrix (breast or liver) fortified with DNC at 200 µg/kg was used as quality control sample (QCs) within every analyzed batch.

**Poultry Litter**

The ground dried sample (2.0 g) was weighed into a 50 mL conical polypropylene tube. After the addition of ACN (20 mL) followed by rapid vortexing, the tube was subjected to an ultrasonic bath (5 min) and rotary shaking (for another 30 min). The solid was settled by centrifugation at 3,500*g* (5 min, 5 °C). An aliquot of the supernatant (800 µL) was transferred to a 15 mL conical polypropylene tube containing 180 mg of Sepra™ C18-E (50 µm, 65 Å, Phenomenex). Ultrapure water (200 µL) was added, and the 15 mL tube was vortexed for 1 minute. The sample extract was filtered on a hydrophilic membrane (0.45 µm) into a 200 µL conical insert (with bottom spring) attached inside a 2 mL vial for HPLC-UV analysis within 24 h. Aliquots of nicarbazin stock solution (at 1000 mg/L) were added on blank litter samples for preparation of matrix-matched analytical curves at 6 DNC levels (10, 25, 50, 100, 150, and 200 mg/kg). These spiked samples were subjected to extraction as well. Blank litter fortified with DNC at 100 mg/kg was used as a quality control sample (QCs) within every analyzed batch.

**HPLC-UV Analysis**

Extracts from feed and litter samples were analyzed on a Thermo Scientific™ Dionex™ UltiMate™ 3000 UHPLC (Thermo Fisher Scientific, Waltham, MA, USA) equipped with a binary pump system (HRG-3400RS), autosampler (WPS-3000), column compartment (TCC-3000SD), and diode-array detector (DAD). A Thermo Scientific Acclaim™ 120 C18 column (150 x 4.6 mm, 5 μm particle size) held at 30 °C was used for chromatographic separations. Mobile phase: consisting of 60% ACN with 40% $NH_4OAc$ 200 mmol/L (v/v) aqueous solution. Flow rate: 0.6 mL/min (53 bar) in isocratic mode. DAD wavelength: 364 nm. Sample compartment temperature: 10 °C. Injection volume: 10 μL. Data acquisition and processing were performed with the Chromeleon™ Chromatography Data System (CDS) software.

**LC-MS/MS Analysis**

Extracts from breast and liver samples were analyzed on a Thermo Scientific™ Finnigan Surveyor Plus LC system (Thermo Fisher Scientific, Waltham, MA, USA) equipped with a quaternary pump system and autosampler. Separations were performed on a Phenomenex Kinetex® C18 100 Å column (100 x 4.6 mm, 5 μm particle size) combined with a C18 guard column. The column temperature was set at 30 °C. A combination of two mobile phases (A and B) was used at a flow rate of 1.0 mL/min (A consisting of methanol and 0.1% formic acid v/v; and B consisting of water and 0.1% formic acid v/v). Gradient mode elution was programmed as follows: 5% A (0-1 min), 80% A (1-2 min), 100% A (2-5 min, held until 7 min), 5% A (7-7.5 min, held until 11 min). The autosampler compartment was operated at 10 °C. Injection volume was 10 μL.

For MS measurements, the LC system was coupled to a Thermo Scientific™ Quantum Access Max mass spectrometer (Thermo Fisher Scientific, Waltham, MA, USA). DNC and DNC-$d_8$ solutions (both 10 mg/L in ACN) were directly infused, at 10 μL/min using an integrated syringe pump, for tuning the MS spectrometer in negative electrospray ionization mode. This exact procedure was also used for ion identification (precursors and their products) and optimization of spray voltage (3.0 kV) and collision energies (CE). For DNC, the deprotonated molecular ion $[M-H]^-$ at *m/z* 301.0 was selected as the precursor ion, while product ions at *m/z* 107.2 (CE 40 eV) and *m/z* 137.1 (CE 21 eV) were set for quantification and confirmation, respectively. The deprotonated molecular ion $[M-H]^-$ at *m/z* 308.7 was selected as the precursor ion for DNC-$d_8$, whose ion product at *m/z* 141.2 (EC 19 eV) was set for quantification. The retention time was used for analyte confirmation as well. With the infusion of DNC and DNC-$d_8$ solutions into the MS spectrometer with mobile phase (50:50 water/ACN both containing 0.1% formic acid) at 1 mL/min, the source conditions were optimized, as follows: vaporized temperature at 261 °C; capillary temperature at 360 °C; sheath gas pressure at 50 psi; and auxiliary gas pressure at 5 psi. Nitrogen was used as the nebulizer gas and argon as the collision gas at a pressure of 1.8 mTorr. Data acquisition and processing were performed using the Xcalibur™ 2.1 software.

**Table S1.** Nicarbazin (NCZ) content in experimental feeds.

| **Growing stage (days)** | **Treatment** | ***N*[1]** | **Formulated (NCZ in mg/kg)** | **Value found (NCZ in mg/kg)[2]** |
|---|---|---|---|---|
| Starter (1–21) | T1/T2 | 10 | 125 | 117.6 ± 19.5 |
| | T3 | 10 | 40 | 34.4 ± 5.2 |
| Grower (22–32) | T1 | 10 | 0 | < LOQ [3] |
| | T2 | 10 | 125 | 120.3 ± 10.3 |
| | T3 | 10 | 40 | 36.5 ± 6.1 |
| Finisher (33–42) | T1/T2/T3 | 10 | 0 | < LOQ [c] |

[1]Total samples collected throughout the experiment. [2]Calculated from the DNC result, considering that NCZ (MW: 426 g/mol) is an equimolar complex consisting of 70% DNC (MW: 302 g/mol) and 30% HDP (MW: 124 g/mol). [3]Limit of quantification (LOQ) = 0.3 mg/kg

**Table S2.** Accuracy and precision for DNC determination in fortified blank matrices used as Quality Control samples (QCs).

| **Matrix** | ***N*[1]** | **Fortified Level** | **Unit** | **Recovery (%)[2]** | **Reproducibility (CV, %)[3]** |
|---|---|---|---|---|---|
| Broiler feed | 6 | 100 | mg/kg | 102 | 7.3 |
| Poultry litter | 16 | 100 | mg/kg | 97 | 9.4 |
| Chicken muscle (Skinless breast fillet) | 25 | 200 | μg/kg | 108 | 14.3 |
| Chicken liver | 18 | 200 | μg/kg | 94 | 12.0 |

[1]Total QCs subjected to analysis throughout the study. [2]Recovery (%) means accuracy;
[3]Reproducibility, expressed as coefficient of variation (CV, %), means precision.

**Table S3.** Levels of probability (*F*-test) from repeated measures regarding interaction effects of treatment, age, and flock on DNC concentration in chicken breast fillet, liver and litter.

| **Cause of variation** | **DNC in breast fillet** | **DNC in liver** | **DNC in litter** |
|---|---|---|---|
| Block | 0.8276 | 0.9848 | 0.0623 |
| Treatment | <0.0001 | <0.0001 | <0.0001 |
| Flock | <0.0001 | <0.0001 | <0.0001 |
| Treatment×flock | <0.0001 | 0.0050 | <0.0001 |
| Age | <0.0001 | <0.0001 | 0.0001 |
| Treatment×age | <0.0001 | <0.0001 | 0.0556 |
| Flock×age | <0.0001 | <0.0001 | <0.0001 |
| Treatment×flock×age | <0.0001 | 0.0023 | <0.0001 |

**Table S4.** DNC concentration in liver from NCZ-fed broilers raised on poultry litter used for multiple flocks.

| Treatment[1] | Age at slaughter (d) | DNC concentration (μg/kg on wet basis)[2,3] | | | | *Pr > F* |
|---|---|---|---|---|---|---|
| | | **Flock 1** | **Flock 4** | **Flock 7** | **Flock 10** | |
| T1 (NCZ at 125 mg/kg fed from 1 – 21d) | 21 | 15281 ± 1119 $^{aA}$ | 11754 ± 1508 $^{bA}$ | 12770 ± 1326 $^{abA}$ | 15138 ± 1306 $^{aA}$ | 0.0251 |
| | 32 | 107.9 ± 17.2 $^{B}$ | 56.0 ± 9.7 $^{B}$ | 115.2 ± 44.8 $^{B}$ | 109.3 ± 15.4 $^{B}$ | 0.9998 |
| | 42 | 24.9 ± 1.1 $^{B}$ | < LOQ | 81.4 ± 18.4 $^{B}$ | 24.5 ± 2.1 $^{B}$ | 0.9357 |
| | *Pr > F* | <0.0001 | <0.0001 | <0.0001 | <0.0001 | - |
| T2 (NCZ at 125 mg/kg fed from 1 – 32d) | 21 | 17311 ± 1117 $^{aA}$ | 12830 ± 558 $^{bcA}$ | 11478 ± 844 $^{cA}$ | 14842 ± 658 $^{bA}$ | 0.0003 |
| | 32 | 13722 ± 822 $^{abB}$ | 12443 ± 1184 $^{bA}$ | 10168 ± 959 $^{cA}$ | 15021 ± 774 $^{aA}$ | <0.0001 |
| | 42 | 122.3 ± 14.7 $^{C}$ | 83.5 ± 16.9 $^{B}$ | 64.3 ± 7.6 $^{B}$ | 53.2 ± 17.2 $^{B}$ | 0.9426 |
| | *Pr > F* | <0.0001 | <0.0001 | <0.0001 | <0.0001 | - |
| T3 (NCZ at 40 mg/kg fed from 1 – 32d) | 21 | 8932 ± 746 $^{aA}$ | 7233 ± 622 $^{abA}$ | 5267 ± 578 $^{bA}$ | 5233 ± 384 $^{bA}$ | 0.0103 |
| | 32 | 4573 ± 349 $^{B}$ | 4324 ± 610 $^{B}$ | 2899 ± 462 $^{B}$ | 4111 ± 598 $^{A}$ | 0.1734 |
| | 42 | 39.0 ± 4.8 $^{C}$ | < LOQ | 48.0 ± 17.1 $^{C}$ | 48.3 ± 13.5 $^{B}$ | 0.9880 |
| | *Pr > F* | <0.0001 | <0.0001 | <0.0001 | <0.0001 | - |

[1]T1: NCZ at 125 mg/kg fed from 1 to 21 d; T2: NCZ at 125 mg/kg fed from 1 to 32 d; T3: NCZ at 40 mg/kg plus maduramicin at 3.75 mg/kg fed from 1 to 32 d; NCZ-free feed (withdrawal period): 22 to 42 d for T1; and 33 to 42 d for T2 and T3. [2]Distinct lowercase letters in the same row differ significantly by the protected t-test ($P \leq 0.05$). [3]Within each treatment, distinct uppercase letters in the same column differ significantly by the protected t-test ($P \leq 0.05$). Values are expressed as the average of 8 replicates followed by the standard-error. LOQ = 20 μg/kg.

**Table S5.** DNC concentration in poultry litter used for rearing multiple flocks of NCZ-fed broilers.

| Treatment[1] | Age at slaughter (d) | DNC concentration (mg/kg on dry basis)[2,3] | | | | *Pr > F* |
|---|---|---|---|---|---|---|
| | | **Flock 1** | **Flock 4** | **Flock 7** | **Flock 10** | |
| T1 (NCZ at 125 mg/kg fed from 1 – 21d) | 1 | NA | 41.43 ± 2.01 $^{a}$ | 23.57 ± 0.52 $^{b}$ | 20.71 ± 1.86 $^{bC}$ | <0.0001 |
| | 21 | 24.10 ± 2.12 $^{bA}$ | 43.64 ± 2.03 $^{a}$ | 28.49 ± 0.94 $^{b}$ | 46.74 ± 3.27 $^{aA}$ | <0.0001 |
| | 32 | 20.15 ± 1.66 $^{bA}$ | 34.76 ± 2.61 $^{a}$ | 25.98 ± 3.20 $^{ab}$ | 30.44 ± 5.60 $^{aB}$ | <0.0001 |
| | 42 | 10.68 ± 1.10 $^{cB}$ | 43.11 ± 5.11 $^{a}$ | 20.73 ± 0.75 $^{b}$ | 24.73 ± 2.18 $^{bBC}$ | <0.0001 |
| | *Pr > F* | <0.0001 | 0.1310 | 0.4880 | <0.0001 | - |
| T2 (NCZ at 125 mg/kg fed from 1 – 32d) | 1 | NA | 88.06 ± 6.34 $^{aA}$ | 41.26 ± 6.29 $^{b}$ | 35.54 ± 3.27 $^{bB}$ | <0.0001 |
| | 21 | 33.73 ± 2.45 $^{dB}$ | 80.06 ± 3.85 $^{aB}$ | 43.75 ± 1.01 $^{c}$ | 60.45 ± 4.06 $^{bA}$ | <0.0001 |
| | 32 | 62.87 ± 3.33 $^{aA}$ | 61.33 ± 1.77 $^{aC}$ | 37.65 ± 8.14 $^{b}$ | 56.10 ± 7.58 $^{aA}$ | <0.0001 |
| | 42 | 32.42 ± 1.45 $^{cB}$ | 74.35 ± 3.19 $^{aBC}$ | 35.03 ± 2.07 $^{c}$ | 56.80 ± 5.66 $^{bA}$ | <0.0001 |
| | *Pr > F* | <0.0001 | <0.0001 | 0.3516 | <0.0001 | - |
| T3 (NCZ at 40 mg/kg fed from 1 – 32d) | 1 | NA | 12.97 ± 0.51 | 13.54 ± 5.34 | 6.57 ± 0.324 | 0.3250 |
| | 21 | 4.42 ± 0.468 $^{bB}$ | 10.59 ± 0.90 $^{ab}$ | 14.24 ± 5.00 $^{a}$ | 14.56 ± 1.65 $^{a}$ | 0.0014 |
| | 32 | 15.00 ± 1.16 $^{aA}$ | 7.75 ± 0.241 $^{b}$ | 9.51 ± 0.669 $^{b}$ | 14.17 ± 1.45 $^{a}$ | 0.0003 |
| | 42 | 6.53 ± 0.683 $^{bB}$ | 11.74 ± 0.75 $^{a}$ | 6.13 ± 0.464 $^{b}$ | 8.00 ± 1.210 $^{ab}$ | 0.0049 |
| | *Pr > F* | 0.0002 | 0.6479 | 0.3713 | 0.2258 | - |

[1]T1: NCZ at 125 mg/kg fed from 1 to 21 d; T2: NCZ at 125 mg/kg fed from 1 to 32 d; T3: NCZ at 40 mg/kg plus maduramicin at 3.75 mg/kg fed from 1 to 32 d; NCZ-free feed (withdrawal period): 22 to 42 d for T1; and 33 to 42 d for T2 and T3. [2]Distinct lowercase letters in the same row differ significantly by the protected *t*-test ($p \leq 0.05$); [3]Within each treatment, distinct uppercase letters in the same column differ significantly by the protected *t*-test ($P \leq 0.05$). Values are expressed as the average of 8 replicates followed by the standard-error. NA = not analysed.

## References


1. Protasiuk, E.; Olejnik, M.; Szprengier–Juszkiewicz, T.; Jedziniak, P.; Zmudzki, J. Determination of Nicarbazin in Animal Feed by High-Performance Liquid Chromatography with Interlaboratory Evaluation. *Anal. Lett.* **2015**, *48*, 2183–2194, doi:10.1080/00032719.2015.1025277.

2. Bacila, D.M.; Cunha, A.; Weber, I.F.; Scheuermann, G.N.; Coldebella, A.; Caron, L.; Molognoni, L.; Daguer, H.; Igarashi Mafra, L.; Feddern, V. Degradation of 4,4-Dinitrocarbanilide in Chicken Breast by Thermal Processing. *J. Agric. Food Chem.* **2018**, *66*, 8391–8397, doi:10.1021/acs.jafc.8b02370.

3. Coleman, M.R.; Rodewald, J.M.; Brunelle, S.L.; Nelson, M.; Bailey, L.; Burnett, T.J. Determination and Confirmation of Nicarbazin, Measured as 4,4-Dinitrocarbanilide (DNC), in Chicken Tissues by Liquid Chromatography with Tandem Mass Spectrometry: First Action 2013.07. *J. AOAC Int.* **2014**, *97*, 630–640, doi:10.5740/jaoacint.13-197.
